# Vertical Transmission of SARS-CoV-2 in Second Trimester Associated with Severe Neonatal Pathology

**DOI:** 10.3390/v13030447

**Published:** 2021-03-10

**Authors:** Gennady Sukhikh, Ulyana Petrova, Andrey Prikhodko, Natalia Starodubtseva, Konstantin Chingin, Huanwen Chen, Anna Bugrova, Alexey Kononikhin, Olga Bourmenskaya, Alexander Brzhozovskiy, Evgeniya Polushkina, Galina Kulikova, Alexander Shchegolev, Dmitry Trofimov, Vladimir Frankevich, Evgeny Nikolaev, Roman G. Shmakov

**Affiliations:** 1 National Medical Research Center for Obstetrics, Gynecology and Perinatology, Ministry of Healthcare of the Russian Federation, 117997 Moscow, Russia; g_sukhikh@oparina4.ru (G.S.); u_petrova@oparina4.ru (U.P.); a_prikhodko@oparina4.ru (A.P.); n_starodubtseva@oparina4.ru (N.S.); a_bugrova@oparina4.ru (A.B.); A.Kononikhin@Skoltech.ru (A.K.); o_bourmenskaya@oparina4.ru (O.B.); a_brzhozovzkiy@oparina4.ru (A.B.); g_kulikova@oparina4.ru (G.K.); ashegolev@oparina4.ru (A.S.); d_trofimov@oparina4.ru (D.T.); v_frankevich@oparina4.ru (V.F.); r_shmakov@oparina4.ru (R.G.S.); 2 Department of Obstetrics, Gynecology, Neonatology and Reproduction, First Moscow State Medical University Named after I.M. Sechenov, 119991 Moscow, Russia; 3 Moscow Institute of Physics and Technology, 141701 Moscow, Russia; 4 Jiangxi Key Laboratory for Mass Spectrometry and Instrumentation, East China University of Technology, Nanchang 330013, China; 201360012@ecut.edu.cn (K.C.); chw@ecut.edu.cn (H.C.); 5 Emanuel Institute for Biochemical Physics, Russian Academy of Sciences, 119334 Moscow, Russia; 6 Skolkovo Institute of Science and Technology, 121205 Moscow, Russia; E.Nikolaev@skoltech.ru

**Keywords:** pregnancy, vertical transmission, fetal growth restriction, COVID-19, coronavirus, SARS-CoV-2

## Abstract

The effects of severe acute respiratory syndrome coronavirus-2 (SARS-CoV-2) infection in women on the gestation course and the health of the fetus, particularly in the first and second trimesters, remain very poorly explored. This report describes a case in which the normal development of pregnancy was complicated immediately after the patient had experienced Coronavirus disease 2019 (COVID-19) at the 21st week of gestation. Specific conditions included critical blood flow in the fetal umbilical artery, fetal growth restriction (1st percentile), right ventricular hypertrophy, hydropericardium, echo-characteristics of hypoxic-ischemic brain injury (leukomalacia in periventricular area) and intraventricular hemorrhage at the 25th week of gestation. Premature male neonate delivered at the 26th week of gestation died after 1 day 18 h due to asystole. The results of independent polymerase chain reaction (PCR), mass spectrometry and immunohistochemistry analyses of placenta tissue, umbilical cord blood and child blood jointly indicated vertical transmission of SARS–CoV-2 from mother to the fetus, which we conclude to be the major cause for the development of maternal vascular malperfusion in the studied case.

## 1. Introduction

Pregnancy is characterized by physiological immunosuppression with predisposition to respiratory viral infections [1]. In previous years, the severe acute respiratory syndrome coronavirus (SARS-CoV) and the Middle East respiratory syndrome (MERS) coronavirus increased the rate of hospitalization in an intensive care unit and lethal outcomes in pregnant women [2]. The effects of SARS-CoV-2 on maternal and perinatal outcomes remain poorly understood due to the limited research of clinical manifestations and laboratory findings in pregnant women with Coronavirus disease 2019 (COVID-19) [3,4]. Thus, there is as yet no consensus regarding the probability and implications of the vertical transplacental transmission of SARS-CoV-2 [5,6,7]. Several findings indicate the possibility of vertical SARS-CoV-2 transmission. First, angiotensin-converting enzyme 2 (ACE2) (the main cellular receptor binding virus) was found to be expressed in the placenta, ovary, uterus and vagina [8]. Second, clinical studies in China revealed immunoglobulin M (IgM) antibodies in neonates from mothers with positive SARS-CoV-2 tests [9,10]. Third, IgM antibodies, representing the acute phase of viral infection, are sufficiently large in size to pass from the mother’s blood through the placenta. Finally, viral RNA and protein were found in the placenta [11,12,13,14]. The vast majority of SARS-CoV-2 clinical cases of pregnant women have been studied in the third trimester of pregnancy. The systematic review by Alexander M. Kotlyar et al. indicated that vertical transmission of SARS-CoV-2 infection in the third trimester is possible but rare (probability around 2–3.7%) and it is not associated with severe neonatal pathology [15]. Out of 936 neonates from mothers with COVID-19, 27 neonates were detected SARS-CoV-2 positive by the polymerase chain reaction (PCR) test with a nasopharyngeal swab. The results of SARS-CoV-2 viral RNA testing were positive for 1 out of 34 neonatal cord blood samples (2.9%), 2 out of 26 placenta samples (7.7%), 0 out of 51 amniotic fluid samples (0%), 0 out of 17 urine samples (0%), and 3 out of 31 fecal or rectal swab samples (9.7%). The results of serology analysis of the neonates based on the presence of immunoglobulin M were positive for 3 out of 82 samples (3.7%) [15]. However, very little remains known about maternal and neonatal outcomes due to SARS-CoV-2 infection in the first trimester and, particularly, the second trimester of pregnancy [11,12,15]. Unlike the first and the third trimesters, the second trimester is associated with notable attenuation of the mother’s immune activity [1]. To the best of our knowledge, only two case reports of second trimester SARS-CoV-2 newborn testing have been published until now [11,12]. In the first case, viral mRNA was found in the placenta and umbilical cord blood of a child born after 22 weeks of gestation [11]. Electron microscopy confirmed the presence of viral capsids on the fetal side of placenta. In the second case, all newborn samples were SARS-CoV-2 negative, except the fetal side of placenta [12]. In both cases, the child did not survive. Acute inflammation in placental tissue was considered to be the main cause of the adverse pregnancy outcome. Further studies are necessary to characterize the effect and potential risks of SARS-CoV-2 infection in the second trimester for fetus development.

Here we report the case of a second trimester pregnancy complicated by SARS-CoV-2 infection at the 21st week of gestation. The complications included fetal growth restriction (1st percentile), right ventricular hypertrophy, hydropericardium, echo-characteristics of hypoxic-ischemic brain injury (leukomalacia in periventricular area) and intraventricular hemorrhage at the 25th week of gestation. The thorough examination of this clinical case indicates the association between SARS-CoV-2 and maternal vascular malperfusion and unambiguously demonstrates vertical SARS-CoV-2 transmission from mother to the fetus associated with severe neonatal pathology.

## 2. Materials and Methods

### Sample Collection and Preparation

All procedures for the collection, transport and preparation of the samples were carried out according to the restrictions and protocols of SR 1.3.3118–13 «Safety procedures for work with microorganisms of the I–II groups of pathogenicity (hazard)». Mothers’ nasopharyngeal swabs and blood, umbilical cord blood, amniotic fluid and the sample of placenta were obtained right before and during C-section. Newborn nasopharyngeal samples were obtained within 3 h after birth. Nasopharyngeal swabs were placed in transport media. Nasopharyngeal specimens were stored at +4 °C and analyzed within 24 h. Blood samples were placed in a tube with EDTA, aliquoted in 100 μL and stored at −20 °C. A sample of placental tissue was obtained from the chorionic side. Autopsy tissue samples of lung, brain, intestine, liver and sample of placental tissues were frozen and stored at −20 °C.

For PCR analysis, tissue samples were thawed and homogenized in 500 μL of RNAase-DNAase-free water. Aliquots of blood and 100 μL of tissue homogenates were pretreated with 400 μL QIAzol Lysis Reagent (Kiagen GmbH, Germany). Virus RNA was extracted from 100 µL nasopharyngeal samples and 200 µL purified homogenates with kit PREP-NA (DNA-Technology LLC, Russia) and eluted in 50 µL.

For proteomic analysis (high-performance liquid chromatography with tandem mass spectrometry, HPLC-MS/MS), frozen tissue (100 mg) was homogenized using a glass-glass tissue grinder in lysis buffer (4% SDS, 150 mM TRIS-HCl, 10 mM DTT, protease inhibitors cocktail). Homogenate was heated at 95 °C for 10 min, sonicated three times for 2 min and centrifuged at 10,000× *g*, +4 °C for 10 min. The supernatant was collected (SDS extract). The pellet was extracted with urea buffer (8 M urea, 50 mM TRIS–HCl) for 30 min at room temperature with constant stirring, centrifuged at 10,000× *g*, +4 °C. Supernatant was collected (urea extract). The protein concentration was determined by BCA assay. Aliquots of each extract containing 100 μg of total protein were mixed with 8 M urea in 0.1 M Tris-HCl, pH 8.5 in the ultrafiltration unit and were then processed by the filter aided sample preparation (FASP) using Microcon 30 k centrifugal ultrafiltration units according to the previous literature [16].

## 3. Pathomorphology and Ommunohistochemistry Examination

Macro and microscopic examination of placenta was performed in accordance with the principles adopted by the Amsterdam placental workshop group consensus [17]. The placenta was weighed without extraplacental membranes and umbilical cord. Tissue fragments (ca. 0.5 cm wide) were excised through all parts of the placenta. The fragments were fixed in 10% neutral formalin and embedded in paraffin. Paraffin sections were stained with hematoxylin and eosin for microscopic examination.

The immunohistochemical study included reactions with polyclonal rabbit antibodies against the S1 subunit of the spike protein (SARS-CoV-2 Spike antibody, GTX135356, GeneTex, USA) and against the nucleocapsid protein (SARS-CoV-2 Nucleocapsid, GTX 135357, GeneTex, USA) with working dilution ratio of 1:500. Immunostaining reactions were carried out on a Ventana Benchmark XT automatic immunostainer with an ultraVIEW Universal DAB imaging system (Roche, USA). For positive and negative controls, sections from SARS-CoV-2 Spike FFPE 293T cell pellet block (GTX435640 GeneTex, USA) and SARS-CoV-2 Nucleocapsid FFPE 293T cell pellet block (GTX435641) were used, as recommended by the antibody manufacturer. The second negative control was done using paraffin sections of tissue from the placenta of a patient without SARS-CoV-2.

For the microscopic analysis of histo- and immunohistochemical reactions and photo documentation we used a light microscope NIKON ECLIPS 80i (Nikon, Japan), morphometry program NIS-Elements AR 5.11 and digital color camera DS-Fi1 (Nikon).

## 4. PCR Analysis

SARS-CoV-2/SARS-CoV Multiplex REAL-TIME-PCR (RT-PCR) detection kit (DNA-Technology LLC, Russia) targeting the N gene and the E gene (specific for SARS-CoV-2) the conserved region of the E gene (common for a group of coronaviruses like SARS-CoV, including SARS-CoV and SARS-CoV-2) was used following the manufacturer’s protocol. The assay includes an internal positive control to identify possible RT-PCR inhibition and to confirm the integrity of the reagents of the kit. Thermal cycling was performed at 35 °C for 20 min for reverse transcription, followed by: (1) 95 °C for 5 min, (2) 5 cycles of 94 °C for 10 s, (3) 64 °C for 10 s, (4) 42 cycles of 94 °C for 5 s, (5) 64 °C for 10 s, (6) 80 °C for 5 s with a thermocycler RealTime system DTprime 4X1 (DNA-Technology LLC, Russia). Any value of the threshold cycle was interpreted as positive for SARS-CoV-2 RNA. Samples were tested twice, starting with RNA isolation.

## 5. Proteomic Analysis of Tissue (HPLC-MS/MS)

The tryptic peptides were analyzed in triplicate on a nano-HPLC Dionex Ultimate 3000 system (Thermo Fisher Scientific, USA) coupled to a TIMS TOF Pro (Bruker Daltonics, USA) mass-spectrometer. The sample volume was 2 µL per injection. HPLC separation was carried out using a packed emitter column (C18, 25 cm × 75 µm 1.6 µm) (Ion Optics, Parkville, Australia) by gradient elution. Mobile phase A was 0.1% formic acid in water; mobile phase B was 0.1% formic acid in acetonitrile. LC separation was achieved at a flow of 400 nL/min using a 40 min gradient from 4% to 90% of phase B.

Mass spectrometry measurements were carried out using the Parallel Accumulation Serial Fragmentation (PASEF™) acquisition method. The electrospray ionization (ESI) source settings were the following: 4500 V capillary voltage, 500 V endplate offset, 3.0 L/min of dry gas at temperature of 180 °C. The measurements were carried out in the m/z range from 100 to 1700 Th. The range of ion mobilities included values from 0.60–1.60 Vs/cm^2^ (1/k0). The total cycle time was set at 1.16 s and the number of PASEF MS/MS scans was set to 10. For low sample amounts, the total cycle time was set to 1.88 s.

## 6. Protein Identification

The obtained data were analyzed using PEAKS Studio 8.5 and MaxQuant version 1.6.7.0 using the following parameters—parent mass error tolerance–20 ppm; fragment mass error tolerance–0.03 Da. Due to the mild denaturation conditions, the absence of reduction and alkylation steps in one of the sample preparation approaches and short hydrolysis time, up to 3 missed cleavages were allowed. However, only the peptides with both trypsin-specific ends were considered for identification purposes. Oxidation of methionine and carbamidomethylation of cysteine residues were set as possible variable modifications. Up to 3 variable modifications per peptide were allowed. The search was carried out using the Swissprot SARS-CoV-2 database with the human one set as the contamination database. FDR thresholds for all stages were set to 0.01 (1%) or lower.

## 7. Results

A healthy 27-year-old primipara at the 21st week of gestation was diagnosed with a moderate form of SARS-CoV-2 infection. Clinical symptoms included hyperthermia up to 39 °C, cough, anosmia, ageusia and decrease in oxygen saturation (SpO_2_) to 92%. According to computed tomography (CT) data bilateral polysegmental pneumonia was detected (15% of lung tissue damage). The patient was administered antibiotics (cephalosporin), low molecular weight heparin, antiviral drugs (lopinavir-ritonavir) and dexamethasone. Oxygen therapy was initiated on day 10. Therapy with low molecular weight heparins was continued until delivery. According to the screening test at 12 weeks three days, nuchal translucency was 1.8 mm, crown-rump length was 59 mm. According to the ultrasound scan at the 19th week of gestation, the fetus size was consistent with gestational age. Amniotic fluid index was normal.

At the 23rd week of gestation when a pregnant woman was already COVID-19 negative and had no clinical signs of disease, the ultrasound scan detected fetal growth restriction (3rd percentile), oligohydramnios (AFI was 2.6), intraventricular hemorrhage, changes in the diffusion of lung parenchyma, hydrothorax, relative cardiomegaly, hyperechogenic bowel. The Doppler scan showed absent diastolic flow in the umbilical artery.

**Figure 1:**
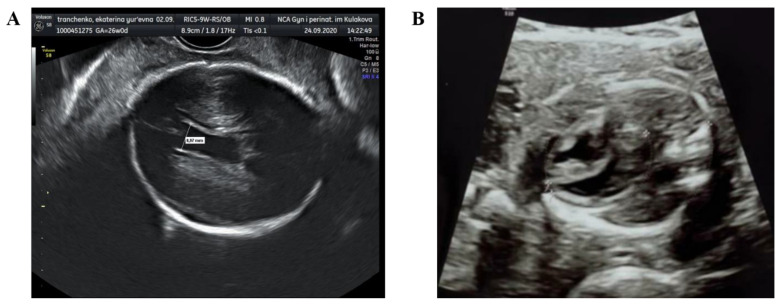
Ultrasound fetal heart scans at the 25th week of gestation: (**A**) intraventricular hemorrhage; (**B**) myocardial hypertrophy.

Starting from the 23rd week of gestation, the fetus was regularly monitored. According to the Doppler scan at the 25th week of gestation the impaired feto-placental circulation was observed—fetal umbilical artery Doppler pulsatility index was 1.9, absent end-diastolic flow, middle cerebral artery pulsatility index was 1.3, peak systolic velocity was 40 cm/s, decreased cerebroplacental ratio was 0.68, a-wave in ductus venosus was positive. The uteroplacental circulation was normal. The ultrasound scan showed fetal growth restriction (1st percentile), right ventricular hypertrophy, hydropericardium, decrease in global heart contractility (Figure 1). According to neurosonography, echo-characteristics of hypoxic-ischemic brain injury (leukomalacia in periventricular area), intraventricular hemorrhage (blood clots in lateral ventricles) and partial agenesis of the corpus callosum were found.

The main clinical parameters of the pregnant woman when admitting to the hospital are presented in Table 1. Urine test showed absence of protein in urine. All parameters in biochemical blood analysis were normal. Markers of angiogenic/antiangiogenic factors PlGF (placental growth factor) 17.08 pg/mL, sFlt-1 (soluble fms-like tyrosine kinase-1) 1846 pg/mL, sFlt-1/P1GF 166.63 demonstrate placental disorders. Screening showed the presence of SARS-CoV-2 antibodies IgG (ELISA kit S-2382 «DS-EIA-ANTI-SARS-CoV-2-G») with positivity index 13.0. IgM antibodies against cytomegalovirus, herpes simplex virus 1 and 2, Epstein-Barr virus were not detected. 

At the 26th week of gestation characteristics of blood flow centralization were detected. We recorded fetal umbilical artery pulsatility index 1.42, positive end-diastolic flow, middle cerebral artery pulsatility index 0.96, peak systolic velocity 46.3 cm/s, cerebroplacental ratio 0.67 (decreased), reverse blood flow in ductus venosus. Ultrasound scan indicated fetal growth restriction (0.1 percentile) and anhydramnios.

Cesarean section was performed at the 26th week of gestation. Premature male neonate was delivered with the birth weight 397 g and length 27 cm. Apgar score at the 1st min and the 5th min was 5 and 7, accordingly. Delayed cord clamping was performed. The neonate was transferred to the neonatal intensive unit (NICU). Neonate examination revealed the congenital pneumonia, disseminated intravascular coagulation, antenatal intraventricular hemorrhage grade 3 on the right side at the stage of cyst formation, congenital anemia and cardiomegaly. The neonate was small for gestational age. Antibodies IgG against SARS-CoV-2 were detected with positivity index 6.3. According to microbiological culture of feces, blood, throat and rhinopharynx did not demonstrate any growth. Asystole was the cause of neonate death after 1 day 18 h 21 min.

**Figure 2:**
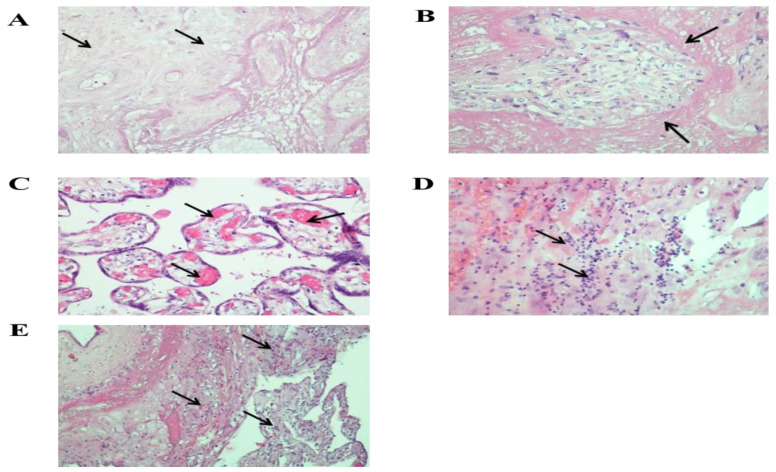
Microscopic changes in the placenta: (**A**)—infarct, ×200; (**B**)—massive deposits of perivillous fibrin, ×200; (**C**)—pronounced plethora of villous vessels, ×200; (**D**,**E**)—lymphocytic-macrophage infiltrate in the decidual tissue of the placenta and extraplacental membranes, ×200; ×100. Stained with hematoxylin and eosin.

**Figure 3:**
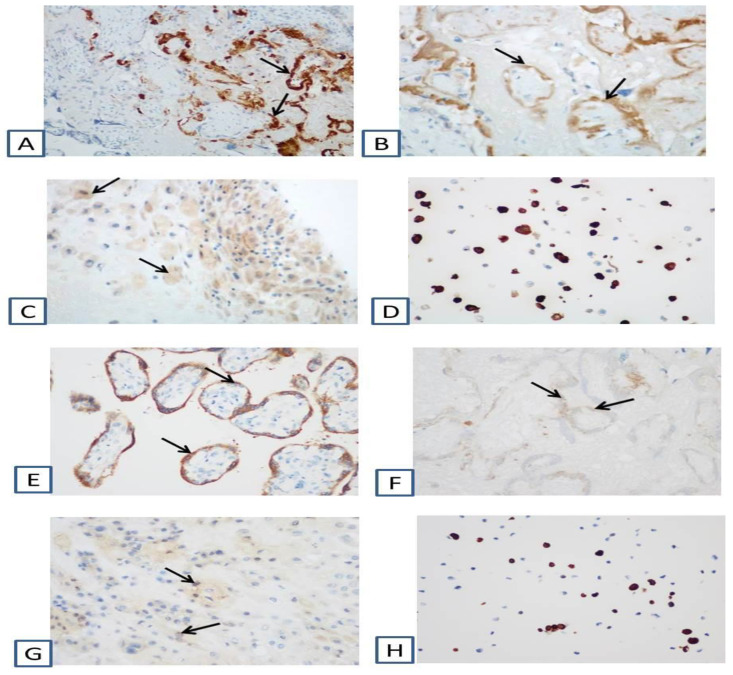
Immunohistochemical changes in the placenta due to the positive immunohistochemical reaction with antibodies against severe acute respiratory syndrome coronavirus-2 (SARS-CoV-2) (Coronavirus disease 2019 (COVID-19)) Nucleocapsid (**A**–**D**) and against SARS-CoV-2 (COVID-19) Spike (S1 subunit) (**E**–**H**): (**A**,**E**)—in the trophoblast of villi, ×200; (**B**,**F**)—in the trophoblast of the villi in the infarction area, ×200; (**C**,**G**)—in decidual tissue, ×200; (**D**)—SARS-Cov-2(COVID-19) Nucleocapsid FFPE 293T cell pellet Block, ×200; (**H**)—SARS-CoV-2 (COVID-19) Spike FFPE 293T cell pellet block, ×200.

According to morphological examination, the size of the placenta was 12 × 9.5 × 1.5 cm, the weight of the placenta was 114 g after separation of the umbilical cord and membranes (less than 10%). On the fetal and maternal surfaces and the incision, extensive areas of old infarct were determined, occupying 1/2–2/3 of the area Supplementary Materials Figure S1A,B. Microscopic examination of the placenta showed numerous old infarcts and large areas of villi surrounded by fibrin (Figure 2). Plethora and hemolysis were revealed in the vessels of terminal and intermediate villi. Small areas of hemorrhage and lymphocytic-monocytic infiltration were found in the decidual tissue. In some areas, neutrophilic leukocytes were determined. In the decidual tissue of the extraplacental membranes, multiple lymphocytic-monocytic infiltrates were observed (Figure 2). The umbilical cord was normal, without signs of inflammation. Immunohistochemical analysis showed strong positive cytoplasmic expression of SARS-Cov-2 Nucleocapsid and SARS-CoV-2 Spike (S1 subunit) in the cytotrophoblast and syncytiotrophoblast (Figure 3).

The corpse of the neonate weighed 470 g (normal weight 739 ± 181 g) and was 27 cm long (normal length 32.2 ± 2.4 cm). The meninges were smooth and shiny. The brain weighed 75 g (normal weight 105 ± 21 g). Brain examination revealed hemorrhage in the lateral ventricles, subependymal hemorrhages up to 0.3 cm in bilateral intraventricular hemorrhage of the 3rd grade with areas of periventricular leukomalacia and hemorrhages in the thalamus Supplementary Materials Figures S1C,D and S2C.

Thymus weighed 0.24 g (normal weight 2 ± 1.1 g), with microscopic signs of accidental involution. Punctate hemorrhages were observed on the visceral pleura. The right lung was 7.27 g, the left lung was 5.8 g. The total lung weight was 19.07 g (normal weight 20.6 ± 6.3 g). The lungs were reddish on the cut. Microscopy analysis revealed canalicular-stage structure, areas of atelectasis, extensive fields of intra-alveolar hemorrhages and the presence of hyaline membranes along the walls of the alveoli (Supplementary Materials Figure S2A,B). The heart was of a cone-shape, size 2.4 × 2 × 1.4 cm, weight 2.77 g (normal weight 5.2 ± 1.3 g). Small punctate hemorrhages were revealed on the epicardium. The valves were formed correctly. The oval window was open with a diameter of 0.3 cm. The thickness of the myocardium of the left and right ventricles was 0.3 cm. Microbiological examination of tissue samples of the lung, liver and blood and intestine revealed no microorganisms.

RT-PCR on the placenta and umbilical cord blood was positive for three SARS-CoV-2 and SARS-CoV-like genes (Supplementary Materials Figure S3).

**Figure 4:**
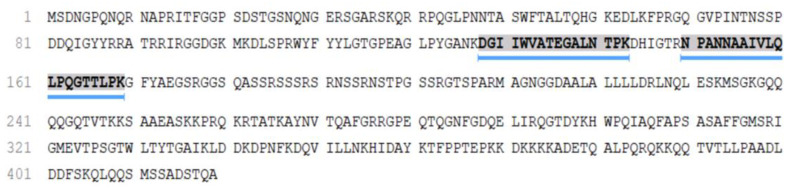
Sequence coverage of the P0DTC9|NCAP-SARS2 Nucleoprotein from SARS CoV-2 in the placenta sample from COVID-19 patient.

Over 1000 proteins were identified in the COVID-19 patient placenta sample, among which the P0DTC9|NCAP-SARS2 Nucleoprotein of SARS-CoV-2 was detected (Figure 4). Nucleocapsid N protein was reliably detected and identified in the COVID-19 patient placenta sample via two unique peptides (Supplementary Materials Table S1 and Figure S4).

## 8. Discussion

Overall, our experience includes 42 SARS-COV-2 positive pregnant women who delivered in the National Medical Research Center for Obstetrics, Gynecology and Perinatology in the period from April to July 2020 [18]. All newborns were SARS-CoV-2 negative according to the results of PCR analysis of the placenta, amniotic fluid, umbilical cord blood, nasopharyngeal and rectal swabs [18]. However, according to the earlier results by Auriti et al., some newborns become SARS-CoV-2 positive on the 5th day, which suggests the possibility of horizontal transmission of the virus [19]. Our experience also includes 62 women who were SARS-COV-2 positive at different stages of pregnancy but recovered by the time of labor. All newborns were SARS-CoV-2 negative according to the PCR analysis. This indicated the absence of transplacental transmission of the virus from mother to the fetus and the teratogenic effect of the virus to the fetus, in agreement with previous studies [20,21]. Previously reported adverse outcomes of the SARS-CoV-2 infection included an increase in the rate of preterm birth and hospitalization of newborns in the NICU [22].

Most studies have found no evidence of vertical transmission of SARS-CoV-2 from an infected mother to the fetus or newborn [5,6,7,23]. However, mostly the infection in the 3rd trimester has been studied until now. Data on the possibility of vertical transmission and the effects of SARS-CoV-19 on the fetus in the 1st and 2nd trimesters remain very limited.

Vivanti et al. were the first to describe a case of delivery at the 35th week of gestation in a woman with the symptoms of SARS-CoV-2 infection, positive PCR result in the placenta, amniotic fluid and in the bronchoalveolar secretions of the newborn. The authors also diagnosed the signs of damage of the white matter of the brain [24]. Also, several reports indicate the association between SARS-CoV-2 infection and the levels of certain molecular receptors, such as angiotensin-converting enzyme 2 (ACE-2). Thus, the levels of angiotensin II and ACE-2 in the placental tissue have been recently reported to increase with the gestational age and indicate the risk of the placental damage in the third trimester [25,26].

Unlike earlier studies, our report describes a case of the second trimester COVID-19 associated with SARS-CoV-2 transmission to the placenta and to the fetus in utero. In this case, our patient had no previous risk factors of severe neonatal pathology, the pregnancy developed normally, as confirmed by the screening tests in the first and the second trimesters. However, two weeks after having experienced SARS-CoV-2 infection, the ultrasound scan detected fetal growth restriction (3rd percentile), oligohydramnios (AFI-2.6), intraventricular hemorrhage, changes in the diffusion of lung parenchyma, hydrothorax, relative cardiomegaly, hyperechogenic bowel. The Doppler scan showed absent umbilical artery flow.

Apart from the SARS-CoV-2 infection, there were no other possible reasons and for the development of such severe placental insufficiency in this woman. Within 4 weeks of dynamic observation, the fetal-placental blood flow deteriorated to critical levels, and therefore a surgical delivery was performed. So it can be proposed that in this case the SARS-CoV-2 infection was the independent risk factor for placental insufficiency and severe neonatal pathology.

The changes revealed in the placenta are consistent with the literature data on the development of maternal vascular malperfusion in SARS-CoV-2 positive women [27,28]. Taglauer et al. and Facchetti et al. observed the increased perivillous fibrin in 46.7% and 26.7% of cases, respectively, and placental infarctions in 33.3% and 40% cases, respectively [29,30]. Therefore, we consider SARS-CoV-2 infection to be the major reason for the development of the placental damage in our study. This conclusion is supported by the results of PCR and mass spectrometry indicating the presence of SARS-CoV-2 in placental tissue and umbilical cord blood. Furthermore, the results of our immunohistochemical analysis show the obvious positive cytoplasmic expression of SARS-CoV-2 Nucleocapsid and SARS-CoV-2 Spike (S1 subunit) in the cytotrophoblast and syncytiotrophoblast, which is a strong indication for the vertical transmission of infection from mother to fetus [24]. This conclusion is further confirmed by the positive results of RT-PCR of the placenta and umbilical cord blood for three SARS-CoV-2 and SARS-CoV-like genes.

Several earlier case reports have shown the presence of SARS-CoV-2 viral RNA and protein in the placenta and virions found within the syncytiotrophoblast [11,12,13,14]. Few studies have found antibodies against immunoglobulin M (IgM) in neonates born from SARS-CoV-2 positive mothers [9,10]. This raises concerns regarding the possibility of intrauterine transmission, as IgM cannot penetrate the placenta.

Also, the developing lesions of the placenta commonly lead to severe disorders of the placenta and are associated with poor obstetrical outcomes such as fetal growth restriction and fetal death [31,32]. Therefore, fetal damage and its subsequent death observed in our study were very likely due to the placental lesions caused by SARS-CoV-2.

The developments of deposits of perivillous fibrin and extensive infarction of villi are due to disorders of the uterine circulation in the placenta. In turn, the deposits of perivillous fibrin and extensive placental infarctions naturally cause fetal hypoxia, which results in bilateral intraventricular hemorrhage and disease of the hyaline membranes. Histological evaluation of the placenta in mothers infected with SARS-CoV-2 has been described in several studies showing various abnormalities [26]. Those abnormalities shared certain pathological patterns, including vascular perfusion failure, fibrin deposition, and chronic willitis or interillosis. In a pathological study of the placenta from a mother infected with SARS-CoV-2, 12 out of 15 placentas showed signs of maternal vascular malperfusion, with 4 placentas showing central and peripheral villi infarctions [28].

Apart from the clinical and morphological value, our results also present the first confirmation of SARS-CoV-2 proteins in infected placenta by proteomics (HPLC-MS/MS). Tryptic peptides identified in the COVID-19 patient placenta sample coincide with the major peptides from our previous study [33]. The N protein, being the most abundant protein in the virion, is the best candidate for mass-spectrometry detection of the SARS-CoV-2. The obtained results confirmed the potential of mass-spectrometry approaches for the detection of the SARS-CoV-2 in different samples including biological fluids and tissues.

## 9. Conclusions

The studied case clearly showed that transplacental transmission of SARS-CoV-2 infection is possible not only in the last trimester of pregnancy, but also in earlier stages of pregnancy. Transplacental transmission can cause the inflammation of placenta and neonatal viremia with the damage of various organs and systems. For the first time, the expression of Nucleocapsid N SARS-COV-2 protein in the placenta was confirmed by proteomic method (HPLC-MS/MS).

## Figures and Tables

**Table 1:** Main laboratory parameters of the pregnant woman.

Parameter, Units	Value	Range
Leukocytes × 10^9^/L	1.41	3.53–42.8
Erythrocytes × 10^12^/L	3.64	2.79–5.26
Haemoglobin g/L	116	75–160
Haematocrit level L/L	0.34	0.34–0.45
Platelets × 10^9^/L	333	91–1058
Lymphocytes %	2.7	5–62
CRP mg	1.28	0.08–229
Fibrinogen g/L	3.59	2.02–9.04
Activated partial thromboplastin time s	28.2	20–38
PR sec	11.3	10.2–20.8
Thrombin time s	21.1	11–16
D-dimer ng/L	1253	25–34,280

## Data Availability

The data presented in this study are available within the article and its supplementary materials at https://www.mdpi.com/1999-4915/13/3/447/s1.

## Supplementary Materials

The following are available online at https://www.mdpi.com/1999-4915/13/3/447/s1, Figure S1: Extensive infarctions of the placenta (A—maternal surface, B—fetal surface, sectional view) and hemorrhages in the brain (C,D), Figure S2: Microscopic changes in the lungs (A,B) and the brain (C): A—hemorrhages in the lumen of the alveoli, g-e ×100; B—hyaline membranes in the alveoli, d ×200; B—hemorrhages in the periventricular region, g-e ×100, Figure S3: Fluorescence cycles in RT-PCR for three SARS-CoV-2 genes (blue, orange and purple color, respectively): 1—the positive control, 2—the placental (A) and umbilical cord blood (B) samples, Figure S4: MS/MS data of detected peptides of P0DTC9|NCAP_SARS2 Nucleoprotein, Table S1: Peptides from the P0DTC9|NCAP_SARS2 Nucleoprotein identified in the placenta sample from COVID-19 patient.
