# Supplementary material for: Vertical Transmission of SARS-CoV-2 in Second Trimester Associated with Severe Neonatal Pathology
Source: Viruses. 2021 Mar 10;13(3):447. doi: 10.3390/v13030447 (PMC7999228; doi:10.3390/v13030447)
Supplement: Supplementary file 1 [file viruses-13-00447-s001.pdf]

Supplementary materials

Table 1. Peptides from the P0DTC9|NCAP-SARS2 Nucleoprotein identified in the placenta sample from COVID-19 patient.

Peptide	–10lgP	Mass	Length	ppm	m/z	z
K.DGIIWVATEGALNTPK.D	93.59	1683.8832	16	2.3	842.9508	2
R.NPANNAAIVLQLPQGTTLPK.G	67.35	2059.1426	20	1.4	1030.58	2

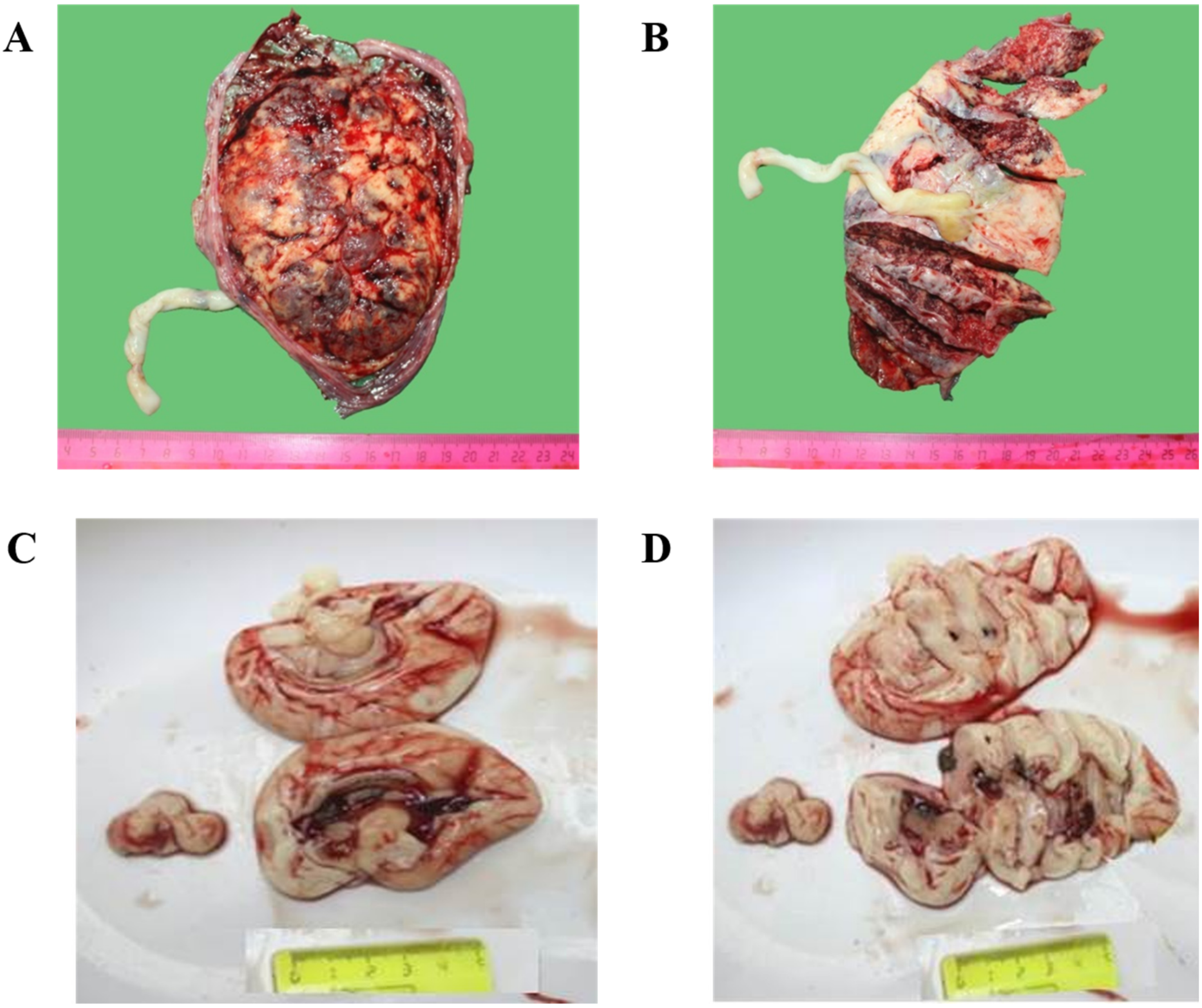

Figure S1. Extensive infarctions of the placenta (A - maternal surface, B - fetal surface, sectional view) and hemorrhages in the brain (C, D).

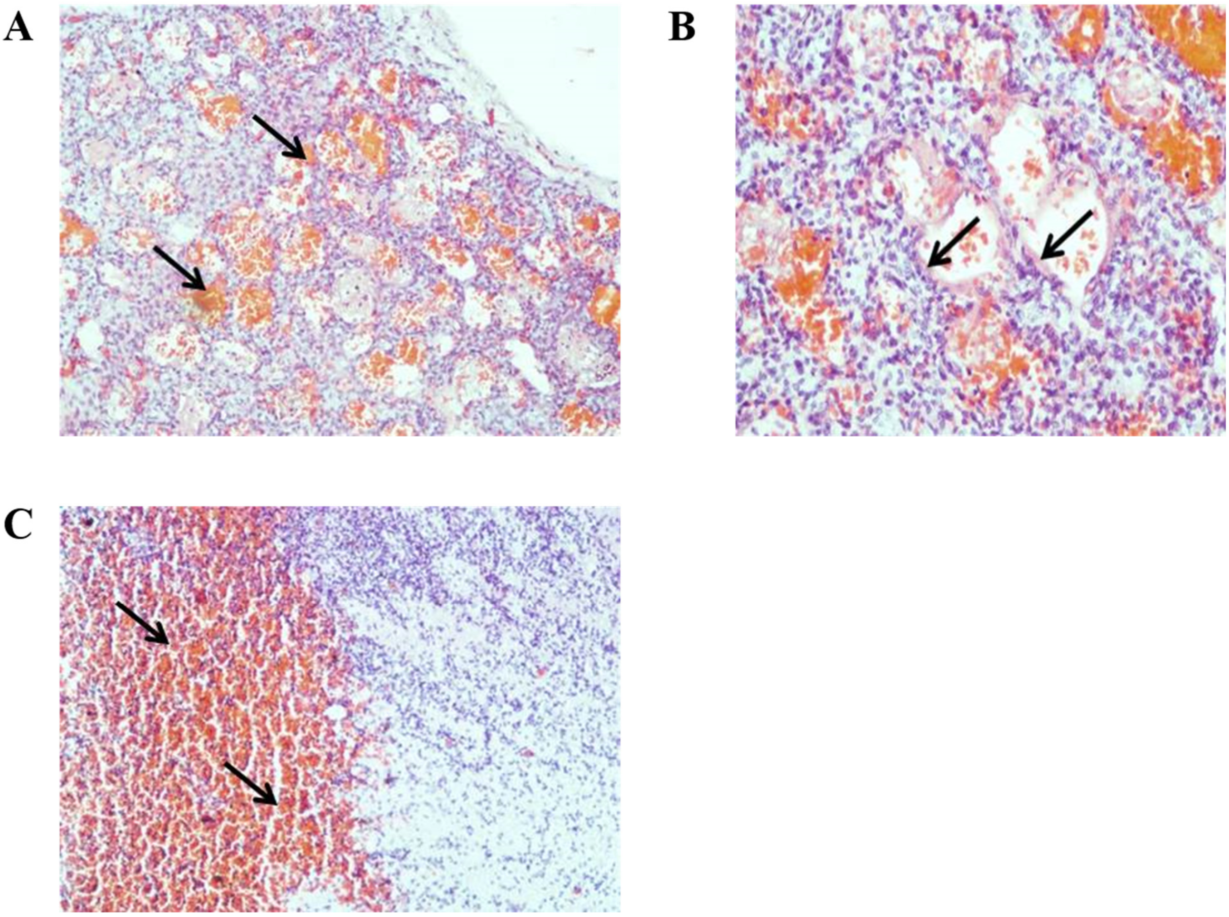

Figure S2.. Microscopic changes in the lungs (A, B) and the brain (C): A - hemorrhages in the lumen of the alveoli, g-e x100; B - hyaline membranes in the alveoli, dx 200; C - hemorrhages in the periventricular region, g-e x100.

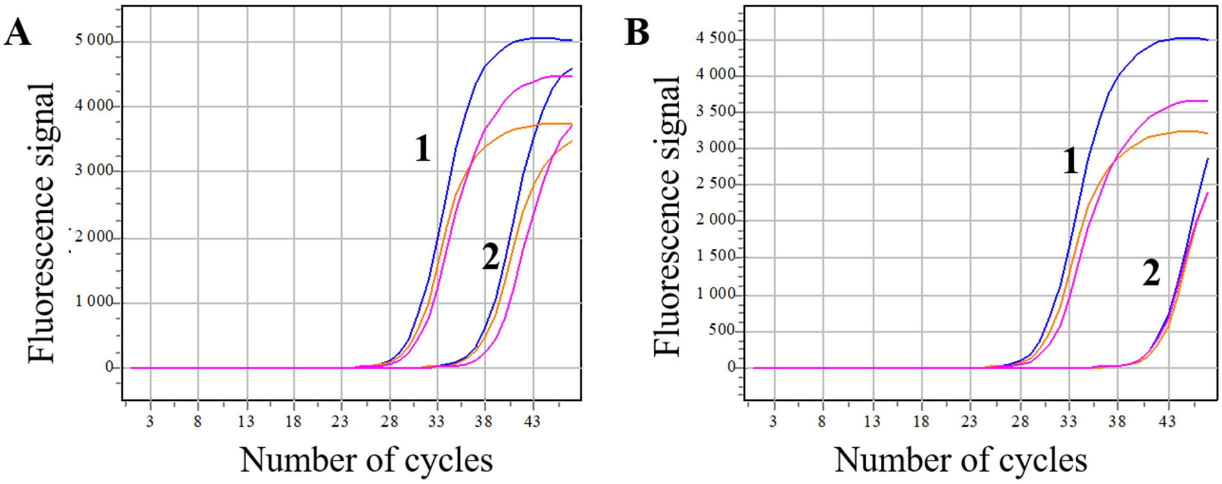

Figure S3. Fluorescence cycles in RT-PCR for three SARS-CoV-2 genes (blue, orange and purple color, respectively): 1 - the positive control, 2 – the placental (A) and umbilical cord blood (B) samples. The earlier the signal is detected, the higher the viral load is.

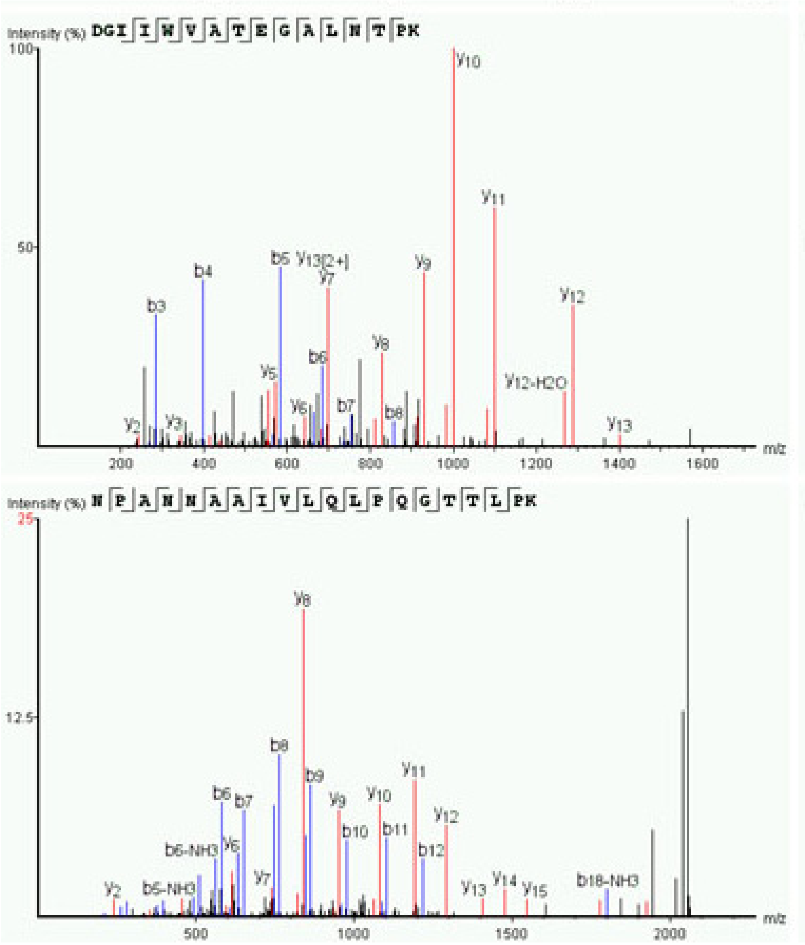

Figure S4. MS/MS data of detected peptides of P0DTC9|NCAP_SARS2 Nucleoprotein.
